# Cancer Risk in Barrett’s Esophagus: A Clinical Review

**DOI:** 10.3390/ijms24076018

**Published:** 2023-03-23

**Authors:** Ahmed Sam Beydoun, Kaleigh A. Stabenau, Kenneth W. Altman, Nikki Johnston

**Affiliations:** 1 Department of Otolaryngology and Communication Sciences, Medical College of Wisconsin, Milwaukee, WI 53226, USA; 2 Department of Otolaryngology-Head & Neck Surgery, Geisinger Medical Center, Danville, PA 17822, USA

**Keywords:** Barrett’s esophagus, esophageal adenocarcinoma, gastroesophageal reflux disease, cancer, RNA sequencing, molecular pathways

## Abstract

Esophageal adenocarcinoma (EAC) is rapidly increasing in incidence and is associated with a poor prognosis. Barrett’s esophagus (BE) is a known precursor of esophageal adenocarcinoma. This review aims to explore Barrett’s esophagus, esophageal adenocarcinoma, and the progression from the former to the latter. An overview of the definition, diagnosis, epidemiology, and risk factors for both entities are presented, with special attention being given to the areas of debate in the literature. The progression from Barrett’s esophagus to esophageal adenocarcinoma is reviewed and the relevant molecular pathways are discussed. The definition of Barrett’s esophagus remains debated and without international consensus. This, alongside other factors, has made establishing the true prevalence of Barrett’s esophagus challenging. The degree of dysplasia can be a histological challenge, but is necessary to guide clinical management. The progression of BE to EAC is likely driven by inflammatory pathways, pepsin exposure, upregulation of growth factor pathways, and mitochondrial changes. Surveillance is maintained through serial endoscopic evaluation, with shorter intervals recommended for high-risk features.

## 1. Introduction

Barrett’s esophagus (BE) is defined by the American College of Gastroenterology (ACG) as intestinal metaplasia (IM) of the distal esophageal squamous epithelium and is a known precursor to esophageal adenocarcinoma (EAC) [1]. Although the true prevalence is difficult to ascertain, approximately two percent of adults are affected by BE, with 0.5–1% going on to develop EAC per year [2]. Gastroesophageal reflux disease (GERD) is thought to be a pathologic driver behind BE, yet 40% of patients diagnosed with BE report no symptoms of GERD at the time of diagnosis [3].

EAC is characterized by a strong male predominance, with a rapidly increasing incidence in the West, now surpassing the incidence of esophageal squamous cell carcinoma (ESCC) in several European, North American, and Oceanic countries [4]. Incidence increases with age, and certain genetic factors may play a role in the development of EAC from BE. The prognosis of EAC is strongly related to the stage at the time of diagnosis; however, due to most cases being diagnosed with the late-stage disease, the prognosis is poor, with a 20% overall survival rate at five years.

The purpose of this review is to summarize the current literature regarding EAC risk in patients with BE and to highlight the unanswered questions regarding these topics for future research.

## 2. Materials and Methods

A literature search of the Ovid Medline/PubMed database was conducted for papers published up to December 2022. The keywords used in the search included combinations of the American and English spellings of Barrett esophagus and esophagus neoplasm or adenocarcinoma. Studies were excluded if they were abstracts, not in English, or not peer reviewed. The references were examined for relevant articles and to ensure a comprehensive review of the literature. The articles were then interpreted and summarized into a cohesive review.

## 3. Definition and Diagnosis

BE is generally defined as columnar metaplasia of the distal esophagus; however, there is controversy concerning the diagnostic criteria for this disease process [5]. This is mainly in regard to whether IM is necessary for the diagnosis. IM is generally characterized by the presence of goblet cells; however, while the presence of goblet cells indicates intestinal metaplasia, not all intestinal metaplastic epithelia contain goblet cells [6]. The ACG, American Gastroenterological Association (AGA), and European Society of Gastrointestinal Endoscopy (ESGE) define BE as a change in the normal esophageal squamous mucosa, greater than 1 cm, that is visible endoscopically and demonstrates IM on biopsy, while the guidelines of the British Society of Gastroenterology (BSG), and Japan Esophageal Society (JES) do not require IM, and BE may be diagnosed with cardiac, intestinal, or oxyntic columnar mucosa on biopsy [3,7,8,9,10].

It is important to consider that differentiating goblet cells from pseudogoblet cells, which occur when columnar cells become distended with mucin and acquire a shape that mimics goblet cells, can be challenging. There is no histochemical or immunohistochemical stains that can reliably identify goblet cells or differentiate them from pseudogoblet cells when they are not apparent on standard H&E stain [11,12]. The difficulty in differentiating goblet and pseudogoblet cells was demonstrated with poor interobserver agreement in a study between seven GI pathologists [6].

In addition to the histologic challenges in the diagnosis, the risk of sampling bias is high. Studies have demonstrated a mosaic distribution of goblet cells within the areas of columnar metaplasia [13,14]. This results in a variation of goblet cell density, and therefore detection rate, depending on the location within the esophagus. The probability of detecting goblet cells has been demonstrated to increase proportionally with the number of biopsies obtained at the time of endoscopy [15]. In an observational study, a minimum of eight biopsies was deemed optimal to diagnose IM in patients with a columnar-lined esophagus on endoscopy. IM was diagnosed at a rate of 68% when eight biopsies were taken compared to 35% when only four biopsies were taken [16]. In addition to a sampling error, the presence and density of goblet cells can fluctuate with time and disease progression. In a study of 43 patients with less than 3 cm of columnar mucosa and no intestinal metaplasia on initial biopsy, Jones et al. demonstrated that 23% of the patients with suspected short-segment (<3 cm) BE had intestinal metaplasia on the repeat biopsy [17]. Kim et al. found that with the repeat biopsy six weeks after study entry and the initial biopsy, 21 (18%) of the 116 patients who met the criteria for BE did so only on one of the two exams [18].

The inclusion of IM in the diagnostic criteria of BE stems from the data supporting a significantly increased risk of dysplasia and EAC in patients with IM. It is argued that IM should be required for the diagnosis until columnar metaplasia without IM is proven to have a significant risk of progression to EAC. Chandrasoma et al. examined 214 patients with columnar metaplasia and demonstrated that IM was present in all 55 cases of dysplasia and/or EAC [19]. A large study of over 8500 patients in the North Ireland Cancer Registry demonstrated a hazard ratio (HR) of 3.54 [95% confidence interval (CI) 2.09–6.00] of developing BE-related adenocarcinoma in patients with IM compared to those without [20]. Advocates for requiring IM for the diagnosis of BE also point to the serious changes in quality of life that result from being diagnosed and labelled with a neoplastic process such as BE [21,22]. An analysis of the University of Chicago Medical Center database over 21 years concluded that the removal of goblet cells from the diagnostic criteria of BE would result in an ~150% increase in BE diagnoses; additionally, none of the patients without goblet cells (*n* = 118) developed EAC or dysplasia, with a mean 5.8 years of follow-up and 2.8 repeat endoscopies [23].

On the other hand, Kelty et al. found a similar risk of EAC in patients with columnar epithelia with and without IM (4.5% versus 3.6%, respectively) in a United Kingdom (UK)-based study, with a median follow-up period of 12 years [24]. Some have argued that sampling could explain the differing results between the Chandrasoma et al. and Kelty et al. studies, with the prior having a rigorous biopsy protocol compared to the latter [5,25]. Takubo et al. classified the mucosa surrounding early mucosal EAC obtained by endoscopic mucosal resection in 141 cases. Overall, 70% of the primary EAC were adjacent to cardiac/fundic-type mucosa as opposed to intestinal-type mucosa [26]. Similarly, Liu et al. found no significant differences in the DNA content abnormalities between samples with and without IM [27].

In summation, due to the concerns with the accurate identification of the presence of IM and the uncertainty regarding whether other mucosal types can progress to dysplasia and EAC, the BSG concluded that the presence of IM is not a defining prerequisite for BE, but that the presence of IM should be taken into account when considering clinical surveillance and follow-up [8]. On the other hand, the AGA and ACG concluded that the diagnosis of BE should include the presence of IM due to the lack of data supporting the risk of the malignant transformation of non-intestinal mucosa [3,7].

EAC is a gland-forming tumor most commonly with tubular, tubulopapillary, or papillary growth patterns. Well-differentiated tumors show >95% gland formation, moderately differentiated tumors show 50–95% gland formation, and poorly differentiated tumors show <50% gland formation [28]. The diagnosis of EAC is typically confirmed via an endoscopic biopsy. Endoscopic ultrasound (EUS) is currently the procedure of choice to determine the depth of the local invasion and clinical T-staging [29]. In a systematic review, EUS had a 91% true positive rate at differentiating T1/2 from T3/4 tumors, which is critical for clinical management [30]. EUS, computed tomography (CT), and fluorodeoxyglucose positron emission tomography (FDG-PET) are the principal modalities for evaluating regional lymph node metastases [29].

## 4. Epidemiology and Risk Factors

### 4.1. Epidemiology

The true prevalence of BE is difficult to estimate due to the sparsity of studies reporting upper esophageal endoscopic findings in the healthy adult population [25]. Multiple studies have attempted to estimate the prevalence of BE, which are summarized in Table 1. Cameron et al. conducted a prospective autopsy study over 18 months, in which 733 autopsies were examined, with seven cases of long segment BE, defined by the authors as an extension of the squamous columnar junction >3 cm above its normal location. The autopsies were predominantly male (61%), and no age distribution was reported. The authors noted that only three of the seven cases had intestinal metaplasia. These findings would suggest that BE was present in approximately 0.4% of the population. This was a significantly higher prevalence than they had estimated by using the number of clinically diagnosed cases from their local shared medical data system [31]. Two European studies in the mid-2000s attempted to estimate the prevalence of BE in the general population through the upper endoscopy of a random population. Ronkainen et al. (conducted in Sweden, *n* = 1000) and Zagari et al. (conducted in Italy, *n* = 1033) both required the presence of goblet cells for the diagnosis of BE, with a prevalence of 1.6% and 1.3%, respectively [32,33]. The study population of Zagari et al. was representative of the general Italian population in terms of age, sex, smoking, alcohol-use, and obesity, except for an under representation of participants younger than 35 years old [33]. The Ronkainen et al. study population was 51% female, with an average age of 53.5 years [32]. The risk factors for BE vary greatly between different populations, making these estimates unlikely to be accurate for any specific population [34,35].

In 2020, esophageal cancer was ranked the seventh highest in incidence and sixth in mortality amongst all cancers worldwide, with over 600,000 new cases and 540,000 deaths [36]. Approximately 70% of esophageal cancer cases occur in men with a 2–3-fold increase in incidence and mortality compared to women [36]. Esophageal squamous cell carcinoma (ESCC) remains the most common worldwide, but there has been a dramatic epidemiologic shift since the 1990s, with the incidence of EAC surpassing that of ESCC in North America, Europe, and Australia [37]. This has been attributed to the decreased rates of heavy alcohol consumption and smoking, and the increasing rates of obesity [38,39]. A landmark paper by Pohl et al. in 2005 demonstrated that the incidence of EAC in the United States (US) increased by six-fold between 1975–2001, and that it was a true increase, as opposed to being due to overdiagnosis or reclassification [40]. EAC cases and deaths in the US are projected to continue increasing until 2030 [41]. Similarly, the incidence of EAC has been projected to rise dramatically during the 2005–2030 time period across high-income countries. By 2030, it is estimated that 1 in 100 men in the UK and the Netherlands may be diagnosed with EAC [42]. In the US, the incidence of EAC increased from 0.4 per 100,000 to 2.8 per 100,000 between 1973 and 2012; 92% of the cases diagnosed during that time were in persons aged >50 years [37].

### 4.2. Barrett’s Esophagus Risk Factors

Clinical, demographic, and lifestyle factors have been reported to affect the risk of BE. GERD has long been identified as the main risk factor for BE, yet 40% of patients do not reports any symptoms of GERD at the time of BE diagnosis [3,8]. On the other hand, an increasing severity and frequency of GERD symptoms has been shown to increase the risk of BE [43,44,45]. Similarly, an earlier age, <30, of GERD symptom onset increases the risk [46]. Studies in human studies, along with rat models, have demonstrated a role for reflux bile in the development of PPI-resistant BE [47]. Many studies have demonstrated an increased risk of BE with hiatal hernias [43,44]. A meta-analysis by Andrici et al. demonstrated the increased risk of BE with hiatal hernia, independent of GERD or BMI [48]. Aspirin has been shown to have an inverse association with developing BE. The use of >325 mg of Aspirin per day was associated with a 0.56 OR of developing BE in a case-control study by Omer et al. [49]. On the other hand, multiple studies have demonstrated Helicobacter pylori positive persons to have a decreased risk of developing BE [46,50,51,52]. The protective effect of H. pylori is hypothesized to be due to decreased gastric acid production and a decreased likelihood of damaging acidic reflux into the esophagus [51]. The current data support white race, male sex, and age > 50 as risk factors for the development of BE [3,43]. A recent meta-analysis of the BE risk factors of over 300,000 individuals demonstrated that the prevalence of BE for various populations was as follows: low-risk general population, 0.8%; obesity, 1.9%; GERD, 3%; age > 50, 6.1%; male sex, 6.8%; GERD in addition to the presence of any other risk factor, 12.2%; and family history of BE or EAC, 23.4% [35]. Smoking has been considered a modifiable risk factor for BE, while alcohol consumption has had mixed results, with some studies demonstrating an increased risk with heavy alcohol consumption (>50 g/day) [53]. Central adiposity has been associated with an increased OR (1.98) of developing BE [34]. The association persisted even after adjusting for BMI.

### 4.3. Esophageal Adenocarcinoma Risk Factors

BE is the precursor lesion to EAC, with a progression from erosive esophagitis to BE, low-grade dysplasia (LGD), high-grade dysplasia (HGD), and finally EAC [54]. Two landmark studies established GERD as a risk factor for EAC in the mid-to-late 1990s [55,56]. The pooled data from five case-control studies demonstrated a 6.24 OR of developing EAC in individuals who had heartburn for at least 20 years compared to those with no heartburn [57]. Studies examining the cancer-protective potential of proton pump inhibitors (PPI) have produced inconsistent results [58,59,60,61]. In 2013, a meta-analysis by Singh et al. pooled seven observational studies with a 71% reduced risk of EAC and/or high-grade dysplasia in patients with BE [59]. Hu et al. performed an updated meta-analysis in 2017 that included two additional studies; however, no dysplasia- or cancer-protective effects were identified in the analysis [60]. Due to these inconsistencies, Chen et al. conducted a metanalysis in 2021 with three additional studies. PPI use was associated with a 0.47 OR of BE progressing to high-grade dysplasia or EAC [61]. Anti-reflux treatment is not limited to medical treatment alone; a meta-analysis of 11 studies revealed evidence that anti-reflux surgery may prevent EAC better than anti-reflux medication, although this effect was only statistically significant when the studies published after the year 2000 (*n* = 4) were pooled. However, the analysis was based on a limited sample size with possible bias and confounding factors [62].

The association between EAC and other medication has also been examined; statins, aspirin, and non-steroidal anti-inflammatory drugs (NSAIDs) have been demonstrated to have inverse associations with the risk of developing EAC [4]. Mechanistically, the protective effect of aspirin and NSAIDs is thought to be mediated through the inhibition of the COX pathways. Immunohistochemical studies have demonstrated COX-2 expression in BE, with an elevation in expression from LGD, HGD, and eventually to EAC [63]. In the general population, a meta-analysis of nine observational studies (two cohort, seven case-control) by Corely et al. demonstrated a protective effect of any aspirin and NSAID use for the risk of developing EAC (OR = 0.67; 95% CI, 0.51–0.87) in the pooled analysis [64]. Stratified analysis was included in the study, aspirin use was protective (OR = 0.5; CI, 0.38–0.66) and NSAIDs had a borderline protective association (OR = 0.75; 95% CI, 0.54–1.0), but EAC and ESCC were both included in this analysis. Similarly, Abnet et al. performed a meta-analysis in which aspirin and NSAID use were inversely associated with EAC (OR 0.64 (0.52–0.79) and 0.65 (0.50–0.85), respectively) [65]. Analysis of the data from randomized controlled trials (RCTs), where the primary outcomes were the prevention of vascular events, provided further evidence for the decreased incidence and mortality from adenocarcinomas with the use of aspirin [66,67]. Furthermore, in a prospective Dutch study of 570 BE patients, NSAID use was associated with a reduced risk of neoplastic progression (HR = 0.47; 95% CI 0.24–0.93) [68]. Statin use was also associated with a reduced risk (HR = 0.46; 95% CI 0.21–0.99). The protective effect was increased with the combined use of both (HR = 0.22; 95% CI 0.06–0.85) [68]. Other case-control studies have also supported the association between statin use and a reduction in EAC risk [69].

Meta-analyses from the late 2000s and early 2010s demonstrated an inverse association between H. pylori and EAC [70,71,72]. A recent prospective German cohort (*n* = 9511) demonstrated similar findings, with a 0.35 hazard ratio of H. Pylori-infected individuals developing EAC during the mean 14 years of observation [73]. Tobacco smoking has been well-established as a strong risk factor for developing EAC. The analysis of the primary data from ten population-based case-control and two cohort studies by Cook et al. revealed that the risk of EAC doubled with ever smoking compared to never smoking (OR 1.96; 95% CI 1.64 to 2.34) [74]. Importantly, this risk does not appear to return to that of never-smokers, even after >20 years of smoking cessation [75]. Alcohol, on the other hand, has not been associated with an increased risk of EAC, even with heavy alcohol consumption (seven or more drinks per day of alcohol) [76]. The meta-analysis and pooled analysis of epidemiologic studies have demonstrated an increased BMI to be associated with an increased risk of EAC in a linear dose-response trend [39,77]. This association does not remain after an adjustment for abdominal obesity. However, abdominal adiposity is independently associated with an increased risk of EAC, even after an adjustment for BMI [34,78].

The association between diet and EAC has been particularly challenging to assess. Due to the relatively low prevalence of EAC, the majority of studies are case-control in design. Such studies are prone to recall bias. The analysis of specific dietary components is difficult due to a high correlation of macronutrients with one another, which suggests that the evaluation of dietary patterns may be more informative than that of individual macronutrients [79]. As there exists a large spectrum of foods and dietary patterns across different population, the results of such studies often have poor generalizability. In an effort to improve the aforementioned limitations, the Factors Influencing the Barrett’s Adenocarcinoma Relationship (FINBAR) study was a population-based study performed in Ireland that evaluated the association between a spectrum of food intake and nutrients with BE and EAC risk within the same population. Fruit consumption was associated with a decreased risk of BE and EAC, while the intake of dietary fat was associated with an increased risk of BE and EAC [80,81]. Three large cohort studies have evaluated diet as a risk for EAC. The NIH-AARP cohort found no association between the total fruit and vegetable intake, total fat intake, and subtypes with a risk of EAC [82,83]. Similarly, vegetable and/or fruit consumption was not associated with a risk of EAC in the European Prospective Investigation into Cancer and Nutrition (EPIC) cohort [84]. The prospective Netherlands Cohort Study was the only of the cohorts to demonstrate a protective effect of vegetables and citrus fruits against EAC, with the protection conferred more in smokers than nonsmokers [85]. Multiple metanalyses of the available observational studies, the majority of which are case-control, have demonstrated a positive association between meat consumption and EAC; however, this association was only present in the case-control studies and no association was demonstrated in the cohort studies [86,87]. In summation, the conclusions regarding the association between diet and EAC are restricted by the limited data from high quality prospective cohort studies.

The discussed risk factors for BE and EAC are summarized in Table 2.

## 5. Progression of BE to EAC

Esophageal epithelia accumulate mutations, chromosomal changes, and multiple clonal populations of cells with age [88]. However, the majority of these mutations are unlikely to drive cancer development due to having only neutral or mildly deleterious functional consequences. Cancer-driving mutations occur in stem or proliferating cells and lead to positive selection and clonal expansion [89]. In discussing the clonal evolution of cancers, the argument of gradualism versus punctuated equilibrium has emerged into consideration [90]. Gradualism describes the gradual evolution of malignant clones through stepwise genetic alterations, with multiple clonal expansions and phenotypic changes. In the case of BE, although there is stepwise histologic progression from nondysplastic BE to LGD to HGD to EAC, the timing between these steps varies greatly between patients [91]. Additionally, biopsy samples of BE have, over time, revealed a low number of stable mutations [92]; this suggests a punctuated equilibrium as an alternative evolutionary model. Punctuated equilibrium refers to pre-malignant clones gradually accumulating mutations over time, without significant clonal expansion or phenotypic change, until one obtains the necessary mutations to enable clonal expansion and sudden phenotypic change [93]. Gradualism and punctuated equilibria are not mutually exclusive and the concurrent occurrence of both is consistent with the heterogeneity of both BE and EAC [79]. The relevance of cancer evolution, clinically, lies in the potential for its use to stratify patients and conduct timely surveillance and early intervention for the highest-risk patients [79].

Pre-cancerous lesions, such as BE, are areas of histologic change associated with an increased risk of cancer. The increased risk of cancer can depend on the specific histologic changes. While BE is the only known precursor lesion to EAC, half of patients diagnosed with EAC in a Mayo Clinic cohort did not have any detectable BE, and the majority of patients with EAC did not have a prior BE diagnosis in a North Ireland Cohort [94,95]. As previously noted in the text, these findings bring into debate whether all cases of EAC arise from BE [26]. In BE, non-dysplastic lesions have been demonstrated in large population-based studies to have a very low risk of progression to EAC (less than 0.3% per year) [96]. This risk increases with the development of LGD and further with the development of HGD.

Histologically, LGD is characterized by nuclear enlargement, cell elongation, hyperchromasia, and stratification with retained nuclear polarity and architecture, while HGD exhibits a greater degree of cytologic atypia and architectural abnormalities [2]. In clinical practice, there remains a high degree of variability and only moderate agreement in the diagnosis and grading of dysplasia in BE [97,98,99]. This was demonstrated in an international study of 51 participating pathologists and a four pathologists reference panel examining 55 esophageal biopsy cases. There was excellent concordance for nondysplastic BE (79%) and HGD (71%), but intermediate concordance for low-grade dysplasia (42%), and indefinite for dysplasia (23%) [100]. Diagnosis can also be particularly challenging in differentiating between LGD and regenerative changes, seen with active inflammation, ulceration, or post-ulcer healing, and between HGD and EAC. Additionally, disagreements exist between pathologists, particularly between those in North America and Europe and those in Japan, in regard to the histologic criteria used to diagnosis and differentiate HGD and early adenocarcinoma [101].

A meta-analysis of 24 studies including over 2600 individuals with LGD reported an annual incidence rate of 0.54% for EAC and 1.73% for EAC or HGD [102]. However, there was significant heterogeneity between the studies. When dysplasia is confirmed by expert pathologists, significantly higher rates of progression have been noted. This is likely due to the overdiagnosis of LGD by general pathologists compared to experienced GI pathologists [103]. In a landmark study, Duits et al. evaluated 293 patients with LGD, in which 73% were downstaged to non-dysplastic BE or indefinite for dysplasia. Endoscopic follow-up was conducted in 264 patients (90%) with a median follow-up of 39 months. For patients with confirmed LGD, the risk of developing EAC or HGD was 9.1% per patient-year. On the other hand, those downstaged to nondysplastic BE or indefinite for dysplasia had a risk of progression of 0.6% and 0.9% per patient-year, respectively [104]. These results are in-line with other studies. In the control arm of the RCT by Phoa et al., which examined radiofrequency ablation (RFA) versus endoscopic surveillance in patients with LGD, 26.5% of the patients developed EAC or HGD over a 30 month follow-up period [105]. Similarly, in a separate RFA RCT, 14% of LGD patients in the control group developed HGD after one year, whilst none developed EAC during that time [106]. HGD is an actionable diagnosis with surveillance not being recommended [3]. The annual incidence of EAC in HGD is reported to be 6%, but studies examining the natural history of the diagnosis are limited [107]. In addition to examining the effect of the presence of dysplasia on the risk of developing EAC, others have examined the extent of the dysplastic. Srivastna et al. studied the long-term outcomes and prognostic value of dysplasia in 77 individuals with dysplastic BE. The individuals were followed for progression to EAC. The extent of LGD was associated with an increased risk of developing EAC, but the extent of HGD was not [108]. This has suggested that once any degree of HGD develops, the individual is at an elevated risk of developing EAC regardless of the extent of the HGD.

The underlying molecular changes that drive this transition remain largely unknown. It has been hypothesized that reflux events and/or inflammatory mechanisms mediate the reactivation of the signaling pathways central to the development of embryonic columnar esophageal epithelium [109]. The Hedgehog pathway has been implicated in this process. The Hedgehog pathway is active during the development of the columnar epithelium of the embryonic esophagus and is normally downregulated in the adult squamous esophageal epithelium [110]. However, the Hedgehog ligand sonic Hedgehog (SHH) is upregulated in BE epithelia [111]. The exposure of squamous esophageal epithelia to the acid and bile components of refluxate induces SHH expression. Clemons et al. have shown that the ectopic expression of SHH induces the columnar differentiation of squamous esophageal epithelium [109,112]. It is thought that SHH expression upregulates the expression of SOX9 via the BMP4-mediated pathways in a paracrine fashion between the epithelia and mesenchyme [109,111]. SOX9 expression is thought to drive columnar metaplasia in the epithelium.

Several other developmental pathways have been shown to play a role in the development of BE, including TGF-beta/BMP, Notch, and Wnt/beta-catenin [109]. The TGF-beta/BMP family of proteins are highly expressed during the embryonic development of multiple organs, including the gastrointestinal tract. Milano et al. have shown that the in vitro exposure of squamous epithelial cells to BMP4 induces the expression of columnar markers [113]. BMP4 is a downstream target of SHH, and the expression of SHH in mouse esophageal epithelium drives the stromal BMP4 expression, which subsequently increases the expression of columnar-specific cytokeratins, likely via SOX9 expression, as previously discussed [111]. The Notch and Wnt/beta-catenin pathways play essential roles in intestinal differentiation [114,115]. In the mouse esophagus, the activated Wnt signaling causes features of dysplasia, but interestingly, not metaplasia [115]. Beta-catenin is upregulated in human esophageal epithelium, which demonstrates HGD, but not in BE without features of HGD. This suggests that the Wnt/beta-catenin pathway does not contribute significantly to the BE pathogenesis, but may play a larger role in the dysplastic changes and malignant transformation [115].

**Figure 1:**
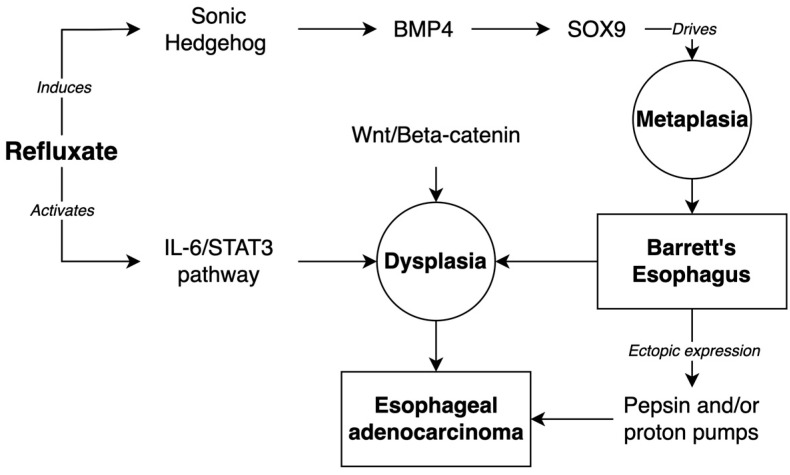
Molecular pathways of development of Barrett’s Esophagus and progression to Esophageal Adenocarcinoma.

The intrinsic and extrinsic inflammatory signaling pathways likely drive the progression of EAC from BE, including the NF-kappaB and IL-6/STAT3 pathways [109]. The inflammation driven by the chronic exposure to gastric refluxate likely induces oxidative stress and the activation of intrinsic inflammatory cascades [116]. Gibson et al. have demonstrated that the IL-6/STAT3 pathway is active in biopsies from patients with BE and EAC, and the increasing IL-6 expression is correlated with the degree of dysplasia in BE [117]. Figure 1 summarizes the discussed molecular pathways.

The specific genetic anomalies that have been suggested to predict the neoplastic progression to EAC in BE include the loss of heterozygosity (LOH) at the loci within 17 p and 9 p, which contain the tumor suppressor genes TP53 and CKDN2A, respectively. The loss of these genes is associated with uncontrolled cell proliferation and tumor progression in multiple cancers. The alteration of CKDN2A has been found to be an initiating event in the pathogenesis of BE, and abnormalities in TP53 are strongly associated with the progression of the neoplastic changes in BE to HGD or EAC [118,119,120]. Interestingly, the LOH at these loci is a frequent occurrence in BE without dysplastic changes, and it is unknown whether they can be used to predict malignant transformation [121].

In addition to specific genetic changes, larger genomic and epigenetic modifications have been shown to play a role in the development of EAC. Aneuploidy has been shown to be predictive of the progression to high grade dysplasia and EAC from BE and the aberrant DNA methylation of cytosine bases in CG-rich sequences (CPG islands), leading to changes in the expression of tumor suppressors, including CDKN2A, which has been shown to be involved in the development of BE neoplasia and EAC [118].

### 5.1. Pepsin and BE/EAC

While the acidic and biliary components of refluxate have traditionally been implicated in the inflammatory processes that drive BE and EAC, recent studies highlight the pathophysiological role of non-acidic pepsin in the development of BE and EAC [122,123,124]. Pepsin and its precursor pepsinogen have been demonstrated to be present in gastric refluxate. Samuels et al. have shown that pepsin exposure to laryngeal squamous epithelium induces cancer-associated changes in these cells [124]. Other studies have demonstrated that the ectopic expression of proton pumps and/or pepsin in BE cells leads to the genetic and molecular signaling pathway changes associated with EAC [125].

### 5.2. Molecular Pathways

The ectopic expression of proton pumps and pepsin in a BE cell line leads to the differential expression of the genes involved in the kinetochore metaphase signaling, tumor microenvironment, and regulation of epithelial-to-mesenchymal (EMT) transition by the growth factors pathways [125]. Dysfunction of the kinetochore metaphase signaling pathway is associated with polyploidy and aneuploidy in dividing cells; Scott et al. have demonstrated the abnormal expression of kinetochore constituents in BE and EAC cells that contributes to chromosome congression failure during mitosis. The tumor microenvironment pathways contain genes important to tumorigenesis, and the EMT pathways regulate the functional transition of polarized epithelial cells to migratory mesenchymal elements, which play a role in metaplasia and metastasis [126,127].

The upstream regulators of these pathways include ERBB2, TNF, and TGFB1. The epidermal growth factor receptor tyrosine kinase ERBB2 is overexpressed in multiple cancers, including breast cancer and EAC [128,129,130]. Interestingly, higher ERBB2 expression is associated with improved survival in patients with EAC [131,132]. TNF is a ubiquitous cytokine involved in inflammation, cell survival, and cell death [133]. Abnormal TNF expression has been associated with BE and EAC [134]. TGFB1 is a secreted ligand of the TGF-B superfamily, members of which are implicated in the regulation of gene expression [135]. TGFB1 is upregulated in BE and EAC and plays a role in EMT [136,137].

### 5.3. Mitochondrial Changes

Transgenic BE cell lines expressing proton pumps, pepsin, or both demonstrate the degradation of mitochondrial cristae, mitochondrial malformations, and the presence of autophagosomes; these changes are consistent with the oxidative stress and aberrant mitochondrial function observed with direct exposure to pepsin [122,138]. These changes indicate alterations in the mitochondrial fusion and fission dynamics, as well as autophagy dysfunction, potentially secondary to the increased mitochondrial reactive oxygen species induced by TGFB1 and TNF [138,139,140]. Excess ROS production has been shown to promote mitophagy in the tumor microenvironment, which in turn increases the resistance to a form of non-apoptotic cell death involving iron known as ferroptosis [141,142]. The genes involved in ferroptosis have been implicated in the pathogenic progression of BE to EAC [125].

## 6. Surveillance

Due to the poor prognosis of EAC, the goal of the endoscopic surveillance of BE is to detect dysplasia or carcinoma at a stage when intervention may be curative. The invasion of the submucosa is considered as that stage by some due to the significantly increased risk of nodal metastasis (9–50% depending on depth of invasion) associated with the EAC invasion of the submucosa [8]. No RCT results currently exist to support the intervals for endoscopic surveillance. Barrett’s Oesophagus Surveillance versus endoscopy at need Study (BOSS) is such a study, taking place in the United Kingdom, examining 3400 BE patients randomized to two year surveillance intervals or “at-need” endoscopy [143]. A meta-analysis and systematic review examining the cohort study evidence of endoscopic surveillance found lower EAC and all-cause mortality, in addition to a higher likelihood of early-stage disease in the surveillance groups compared to those with incomplete or no surveillance. However, adjustment for lead- and length-time biases eliminated or attenuated the beneficial results [144]. Similarly, case-control studies have demonstrated no mortality benefit to surveillance [3]. Hence, the current evidence supporting a mortality benefit to surveillance is weak. Considering the variable annual cancer conversion rate between varying forms of BE dysplasia, it has been suggested that endoscopic surveillance should be performed at intervals based on the degree of dysplasia noted on the previous biopsies [3]. Additionally, the risk for the progression of BE to HGD or EAC has been found to increase with the increasing segment length on meta-analysis [145]. This has prompted guidelines from Europe, the United Kingdom, Australia, and, more recently, the United States to stratify surveillance intervals based on the length of the BE segment [3,8,9,146]. Although there is insufficient evidence to be recommended by the various guidelines, biomarkers such as TP53 have been utilized for risk stratification. In a recent study by Redston et al., p53 immunohistochemistry enabled the identification of higher-risk patients earlier and more frequently than a diagnosis of LGD or indefinite for dysplasia [147].

## 7. Conclusions

This review provides a summary of our current understanding of the definition, epidemiology, and risk factors of BE and EAC. The cellular and molecular changes that drive the progression of BE to EAC are explored. Despite continual advances in our grasp over these topics, several considerable challenges remain. A uniform definition of BE is needed at an international level to provide consistent and reliable data for future studies. Relevant to that is the continued debate regarding the risk of the neoplastic progression of non-goblet cell columnar epithelium. Diagnostic challenges remain for both BE and dysplasia, with no clear histologic or immunohistochemical markers for more reliable diagnoses and differentiation. Additionally, there are no established markers for the prediction of neoplastic progression, and further work is needed to explain the variable timescales in the progression from BE to EAC. Finally, additional data are needed to establish evidence-based intervals for endoscopic surveillance.

## Figures and Tables

**Table 1:** Summary of discussed studies estimating Barrett’s Esophagus prevalence.

Authors	Year of Publication	Study Population (*n*)	Method of Diagnosis	Estimated Prevalence
Cameron et al. [31]	1990	Tertiary center unselected autopsies (733)	Autopsy	0.4%
Ronkainen et al. [32]	2005	General population-Swedish adults (1000)	Endoscopy	1.6%
Zagari et al. [33]	2008	General population–Italian adults (1033)	Endoscopy	1.3%

**Table 2:** Risk factors for development of Barrett’s Esophagus (BE) and Esophageal Adenocarcinoma (EAC).

Factor	Barrett’s Esophagus		Esophageal Adenocarcinoma	
	Association [Reference]	Comments	Association [Reference]	Comments
Gastroesophageal reflux disease (GERD)	Positive [3,8,43,45]	Increased risk with increasing frequency and severity of symptoms	Positive [55,56]	Increased risk with longer duration of heartburn (>20 years)
Hiatal Hernia	Positive [48]	Increase in risk is independent of GERD and Body-Mass Index (BMI)	-	-
Aspirin	Negative [49]	Use of >325 mg of aspirin daily was associated with 0.56 odds ratio of developing BE in a case-control study	Negative [64,65,66,67]	Meta-analysis of observational studies with 0.64 odds ratio of developing EAC with any aspirin use. RCTs examining primary outcomes of vascular events have also supported this association.
Non-steroidal anti-inflammatory drugs	-	-	Negative [64,65,66,67,68]	Association demonstrated by observational studies
Statins	-	-	Negative [68,69]	Dose and duration dependent decrease in EAC incidence with statin use has been demonstrated in some studies
*Helicobacter pylori*	Negative [46,50,51,52]	Hypothesized to be due to decreased gastric acid production	Negative [70,71,72,73]	0.35 hazard ratio of infected individuals to develop EAC in a cohort with a mean observation of 14 years
Smoking	Positive [53]	-	Positive [74,75]	Association persists even after >20 years of cessation
Alcohol Consumption	Mixed [53]	Self-reporting questionnaire with strong positive association with heavy alcohol consumption (>50 g/day)	None [76]	No increased risk of EAC even with heavy consumption (>7 drinks/day)
Central adiposity	Positive [34]	Increase in risk is independent of BMI	Positive [34,78]	Increase in risk is independent of BMI
Proton pump inhibitors	-	-	Mixed [58,59,60,61]	Most recent meta-analysis with 0.47 odds ratio of progression of BE to high-grade dysplasia or EAC
Anti-reflux surgery	-	-	Negative [62]	Meta-analysis limited by included studies’ sample size

## Data Availability

No new data were created or analyzed in this study. Data sharing is not applicable to this article.
